# Dental Bacteriology

**Published:** 1897-09

**Authors:** 

**Affiliations:** Saginaw


### Dental Bacteriology

BY DR. LOEFFLER, OF SAGINAW.

Read before the Michigan Dental Society.

Bacteriology or rather dental bacteriology has finally been thoroughly recognized as an essential part in the dental curriculum; because with such eminent men as Dr. Miller, of Berlin, at its head, it has done more to explain the true nature of the various diseases of the oral cavity than any of the other sciences required in a typical dental education.

It is hardly necessary at this time to indulge in any lengthy arguments in the way of citing historical facts concerning the rise and progress of this wonderful science. Formerly many of the facts relating to this subject although well supported by the masterly deductions of such men as Henle, Rindfleisch, Waldeyer and others, were destined to meet with violent opposition with many of the most prominent men in the medical profession. These men were brought up in other beliefs, and were in no great hurry to cast them aside.

Earnest workers, such as Hansen, Friedlander, Frankel, Dock and many others interested in this field of work were not prevented, however, from accummulating great masses of evidence in favor of the new doctrine, until at the present time no one would have the courage to deny the fact that the study of bacteriology has brought about a revolution in dental as well as medical science within the last ten years, such as has not taken place since the time of Hippocrates.

But even after this point was reluctantly admitted and bacteriologists had scored a most decisive victory over all opponents, a new plan of opposition was brought out. "It is all very well for those who are interested in such things to know that this micrococcus is the cause of this disease, and that bacillies the cause of another; but what good is to be derived from such knowledge so long as it does not help us to treat the disease more successfully."

This argument is altogether too short-sighted to admit of any discussion, since it must be admitted by every one that to know the cause of a trouble vastly increases the possibility of discovering a cure.

Moreover, the inspiration given to the study of bacteria in the last few years, cannot fail to bring forth its fruits, and I am convinced that it is only a question of time when the intelligence of men shall triumph over the malignity of these lowest forms of vegetable life.

Under any and all circumstances, the fact is incontrovertable that nine-tenths of the important discoveries in the domain of pathology, which have been made during the last ten years must be ascribed to bacteriology and any medical or dental practitioner, who to-day is not informed of this branch of medicine, is lamentably deficient at a most vital point in his professional requirements.

The dentist of to-day, as a member of a learned profession, as a practitioner of a speciality of medicine or simply as an educated man, cannot afford to be without a knowledge of those things, which have done and are still doing so much to revolutionize the whole science of medicine.

The purpose of this communication is not solely to point out the great advancement that has been made along these lines, but to create a stimulation for greater activity in this domain of dental science, especially among the members of this association.

We are often very willing to listen to those who have gone before us, and secure if we can the prestige of their authority for our opinions; but what we really value is temporary help. My impression is, if this society would spend more time in seeking to bring out the talent which is in its own membership, and only occasionly call on outside help, we would do better work. The average member of any society does not care to assume that he is fully equal to discussing a paper, which he has never heard before, and which has consumed many weeks in its preparation, unless it be some one who does not carry his sensibilities too close to the surface.

The fact has so impressed itself upon my mind that I have deemed it highly necessary to mention it in this connection, and trust that its true import may be fully realized in the further consideration of the subject before us.

We have, to a certain extent at least ample facilities for prosecuting this work relating to the nature and function of bacteria. We have for example that marvelous production on "The Micro-organisms of the Human Mouth," by Dr. Miller, of Berlin, and which, by the way, ought to be found in the library of every energetic dental practitioner of to-day.

The microscope is also within our reach. In case we have not one of our own we can borrow one from our neighbors.

Concerted action on the part of the different members of the Michigan State Association would go far toward encouraging the work that is being done in our State University. How greatly the interest in any particular subject is magnified to those who have given it special attention even for one year! We are, in some respects at least, too much dependent upon what others have said and worked out for us, especially in regard to scientific subjects. No matter what a Pasteur, a Koch or a Miller have given out as their experience or verdicts—we all want to discuss the question from our own point of view, without preferences, prejudices, difficulties and desires.

But a short time ago I heard a remark by one of our fellow practitioners that would not have been made, had there been a little more thorough investigation in this direction. In other words, he said, "That as far as he was informed there had been no material progress made in bacteriology for the last twenty years." The trouble was, his information on the subject did not go very far.

The many points of interest and beautiful sights revealed to us by the aid of the microscope, cannot be fully appreciated until this wonderful instrument has demonstrated these facts to our own eyes.

The amount of practical work I have done in this direction, though limited, has been a great help to me. It consisted largely in the examination of the mucus, saliva and deposits as found in the mouths of various patients. A number of attempts were also made to prepare sections of carious teeth, but in every case it has been a failure, which was largely due to the fact that we had neither the skill nor the facilities for doing that kind of work.

The study of bacteriology ought to be carried on in a systematic way. Special attention ought to be given to the morphology and classification of micro-organisms, especially the principal forms of bacteria as we find them in the human mouth, and in connection with the conditions under which they are capable of proliferation.

The most essential part of the work, however, seems to be to make a thorough study of the physiology and chemistry of those processes going on in media in which bacteria exist, (fermentation, putrefaction,) and the products resulting from them. At all times, of course special attention ought to be given to bacteria of the human mouth and teeth; their action upon dead organic matter found in the mouth as well as upon the teeth, mucus membrane, etc.; and their eventual action upon more remote parts of the body; In particular, their relation to diseases of the digestive tract, lungs, and to pyemic and septicemic processes. And lastly to the dangers of infection accompanying operations in the human mouth, and the precautions to be taken for avoiding them.

To accomplish all of this, especially the practical part of the work, is not all clear sailing, but has its usual amount of difficulties to be overcome the same as in any other undertaking.

We are sadly in need of advancement in the way of prophylactic treatment of the teeth, and in no way can this be better accomplished than by obtaining a more thorough and general understanding of the nature of the processes going on in the human mouth, and of the means available for combating them. There is a question in regard to this subject which is not so generally understood, and one that I wish might be fully discussed, and that is: Which is first, the bacteria or the fermentation?

Dr. Miller and many other equally good authorities, think it is actually astonishing that this question can still claim the attention of any mind, and that irrefutable facts can be overlooked, especially where there are so many and valuable text books of bacteriology in which ample information may be found in regard to these vital questions.

Many dentists of marked reputation claim that acid may be formed directly from the putrefaction of organic substances in the mouth, irrespective of micro-organisms, and obstinately deny that they are at all necessary.

Those, however, who are at all familiar with the essential principals underlying the subject of bacteriology, will agree that the process of fermentation and putrefaction are caused by the presence of living micro-organisms.

Dr. Miller has established this fact, it seems to me, beyond the shadow of a doubt. His experiments are conclusive and show plainly that we have to do with a ferment which is capable of reproducing itself, in other words, the agent giving rise to acid fermentation in the juices of the oral cavity exist in the form of living organisms.

From time to time during the discussion of the relations of micro-organisms to the process of fermentation, there have appeared suggestions that decay of the teeth might be the result of the action of microbes. There is a mistaken idea, and one which is still entertained that micro-organisms, in order to be instrumental in the decay of teeth, must first enter the substance of the tooth. This conception, however, seems to be dependent upon a failure to fully comprehend the theory of fermentation with the production of an acid, which being formed, in contact with a particular part of a tooth acts chemically upon its substance and decomposes it. This fact should at all times be remembered, that, if microbes act at all in this process, it is through their agency in setting up fermentation, during the process of which substances are so changed chemically, as to produce an acid, or some other agent capable of acting upon the structure of the tooth and decomposing it. Therefore contact with a certain portion of a tooth, provided this be sufficiently prolonged, is all that is required.

The chemical substance or acid provided, goes before and prepares the way for the entrance of fermentive agents. This fact, being kept in mind, much of the confusion of ideas apparent in the earlier discussions of this question, will be prevented.

The work of Leber and Rottenstein made a profound impression on the dental profession, notwithstanding the fact that most of their propositions fell from lack of evidence. The subject has since been taken up by others, improved methods have been devised and our knowledge has been greatly increased. In this work Messrs. Miller and Underwood, of London, have taken an important part. Their results were communicated at the meeting of the World's Medical Congress, held in London, in 1881. These gentlemen had the use of the improved staining methods introduced by Dr. Koch, and fully verified the findings of Leber and Rottenstein as to the presence of micro-organisms in the tubules of carious dentine. After an extended series of flask experiments, they announced the conclusion that the decalcification of the teeth in decay is accomplished by an acid secreted by the organisms, and that there are other phenomina present, such as the widening of the tubules and the discoloration of the decayed mass, which are not to be explained by the action of acid alone. The widening of the tubules is regarded as being the direct work of the micro-organisms, which consume the dentinal fibres and the decalcified walls of the tubules, and in this way finally break down the entire mass.

In regard to the relation of micro-organisms to the phenomena as observed in caries of the teeth, the conclusion of these observers was an advance upon anything before developed, and considering the fact that they were not able to make out any of these processes by any manner of demonstration, it seems quite remarkable that they should have approached so nearly to accuracy of judgment. They did not make the necessary separation of organisms, nor study their individual characters and the changes they produced when in contact with different substances.

We are under many obligations to Dr. Miller, who has done this work in the case of at least some of the micro-organisms of the mouth, and it only requires a continuance of the work to make us familiar with them all. Any amount of energy has been expended in other directions; may we not use an equal amount along this line of investigation, and to very good advantage?

The members of this association can do a vast amount of good by making one united effort in this direction. For how can we better prepare ourselves for treating disease than by making ourselves thoroughly acquainted with its cause, the manner in which it operates, the conditions essential to its action, the means by which it may be rendered harmless?

In conclusion I might say that Dr. Miller's work on "Micro-organisms of the Human Mouth," is within our reach, and may be had from the S. S. White Dental Mfg. Co. for $5.00. This work being once in your possession the practical part of the work with the microscope is sure to follow.

DISCUSSION.

Dr. Codding: Being a firm believer in bacteriology it would be impossible for me to disagree with Dr. Loeffler. There is but one question advanced by Dr. Loeffler that needs to be discussed and it seems to me that, that matter is pretty well settled at the present time, though I am well aware of the fact that a few years ago a few here were not very firm believers in bacteriology. Dr. Loeffler wished it discussed whether fermentation preceeds bacteria or vice versa. Dr. Miller says that fermentation is the direct result of bacteria. I have read Dr. Miller very carefully and if any of you have not, I think you could not invest five dollars in any work that will repay you as fully, as a work that treats of the micro-organisms of the mouth. After the acid is formed which destroys the enamel, then the bacteria have their own way. Dr. Miller says that in every mouth he has examined he has found five bacilli to one bacteria, and that in eighteen of them, ten gave an acid reaction. From the fact that much of our food consists of carbo-hydrates, we can readily see why decay is produced. Dr. Miller took saliva and mixed it with starch, and at the end of five hours had an acid. He sterilized the starch and had the same result.

After sterilizing the saliva he had no acid which would demonstrate that the acid reaction destructive to enamel, is due wholly to the bacteria which have this acid reaction. I have been asked of what use was a knowledge of bacteriology to dentists. It has been of great use to me. I will tell you how. A dentist knowing what causes decay, as we can ascertain from Dr. Miller's work, will advise his patients, and the mother or parents of his younger patients, to see that their teeth are thoroughly cleaned. That these substances are removed, which can be done by brushing and by the use of some antiseptic. Dr. Miller has a list of about thirty or thirty-five antiseptics that can be used. It was a surprise to me that one we have used in the office a great deal was spoken of as the best, that is listerine. It will totally devitalize all the micro-organisms of the mouth in one-half minute. Oil of peppermint will in several minutes. So we can see that if the patients are instructed to thoroughly cleanse the mouth, to remove as many as possible of these germs, there will be much less cause for decay. The idea has been advanced that meat causes decay. There never was a greater mistake. The action of the meat is alkiline. The Greenlanders and Laps and Fins have the finest teeth of any race. Their diet consists of meat and fish. The percent of decay of the teeth of these people is but four percent. The percent was greatest among those who live upon starchy foods. Another point of action for bacteria is upon the gums. Many of you have had patients whose gums looked raw. What is the cause of that? We ought to know and be able to prescribe a cure. Accumulation of the debris of carbo-hydrates being deposited upon the gum, forms a genial soil for the bacteria. Those gums can be restored to a healthy condition by thoroughly freeing the teeth of all deposits of tartar, and instructing the patient to use the tooth-brush before retiring. You will be surprised at the result in two weeks.

DR. HOFF: You have already had a very elaborate exposition of this subject. I am not going to take the opposite side. I heartily concur with the remarks and with the paper and they are entirely pertinent to the time. A few years ago the subject of bacteria was the important one. To-day cataphoresis; to-morrow it will be something else. Bacteriology is here to stay. We did not know formerly when we were using antiseptic drugs and advocating cleanliness, that we were doing it on bacteriological principles. The old-time dentist who had nothing but a creosote and iodine bottle, to tell the truth, had a pretty fair outfit. The more experience I have with creosote, the more I am convinced there is no better germicide nor antiseptic either. I think in a few years our offices will again take the creosote odor. There were several statements made in the paper, and by Dr. Codding, in regard to the cause of caries. Most of us are pretty well convinced that dental caries arises from the activity of bacteria. The old idea was that decay was caused by the mineral and other acids generated in the mouth. That they encouraged and started decay of the teeth. I don't know but that there is a good deal in that theory. Dr. Watt's theory was entirely a chemical one. That decay was the result of a chemical reaction.

The result is the same. Dr. Codding says that meat products do not cause decomposition. Dr. Watt's says that meat causes nitric acid decay which is the most destructive. His theory was that ammonia was formed in the decomposition of the meat and from the air passing through the mouth, oxygen was taken by the ammonia resulting in nascent nitric acid, which would destroy tooth tissue more rapidly than any other. His theory is supported by the fact that nitric acid is found in the mouths of persons who breathe through the mouth. It seems to me that it is entirely possible that such chemical action does take place in the mouth and that decay is not entirely confined to results of the action of acids which are developed by micro-organisms. These two theories may be altogether harmonious. They may not be at so much variance as our bacteriological friends would have us believe. While I don't entirely uphold Dr. Watt's theory, I believe there are elements of truth in it, and it explains many of the phenomena we have in the mouth. Still I feel I can take a liberal position. We ought to take it into account in the diagnosis of the diseases we find in the mouth, in prescribing, and in instructions we give our patients with regard to the care of their teeth. I don't think it is essential that we use such strong germicides as Dr. Miller generally recommends. The listerine recommended by Dr. Codding, and other newer preparations we have out in the display rooms, are all excellent in their way but I do not think they are necessarily essential. A good tooth-brush, a tooth-pick of proper material, a bland soap and some warm water, will do as much good if properly used, as any other germicides. Listerine is entirely soluble. While it may sterilize the mouth for two minutes, how long will it remain sterilized? It has been found that after keeping bacteria in bicloride of mercury that they can be resuscitated, is it possible to so sterilize the mouth by the use of these antiseptics that we will produce any beneficial results to our patients? It is the practical application we must look for and we must study it from the bacteriological standpoint. I think we can occasionally use them to a very good advantage, but we cannot say to our patients that by using them five times a day, that his teeth will be protected from further decay. It is the same principle that we have all found in connection with sterilizing our instruments. We thought a few years ago that we must use bicloride to sterilize every instrument. We have been hunting for something ever since, because we found that it destroyed our instruments. My idea is that the old-time principles of cleanliness will do more to sterilize our instruments practically than by using so many chemical disinfectants and making the instruments look so badly that patients hesitate before they will let you use them. Ordinarily a hot water bath will suffice for all practical purposes. I don't think it is necessary to have a bicloride bath or any other kind of a bath, which ruins the cutting edge or renders the instrument unsightly.

We have got to use practical common sense, the same as with cataphoresis. There are none of us who would sterilize our clothing. We would send them to the washer-woman and feel confident that they are clean enough. I think the same principle with regard to the mouth would be equally acceptable. I don't wish to be understood to say, that in cases where we know we are dealing with infectious disease, that we should use the same instruments on other patients without thoroughly sterilizing them. The idea of sterilizing every burr, every chisel, every excavator, every time they may be used, it seems to me a needless waste of time. We should study this subject; make ourselves masters of it. We want to know how we can use it to our advantage and the welfare of our patients.

